# Is adjustment for reporting heterogeneity necessary in sleep disorders? results from the Japanese World Health Survey

**DOI:** 10.1186/s12888-016-0733-9

**Published:** 2016-02-06

**Authors:** Md. Ismail Tareque, Nayu Ikeda, Atsushi Koshio, Toshihiko Hasegawa

**Affiliations:** Department of Population Science and Human Resource Development, University of Rajshahi, Rajshahi, Bangladesh; Center for International Collaboration and Partnership, National Institute of Health and Nutrition, National Institutes of Biomedical Innovation, Health and Nutrition, Tokyo, Japan; The Graduate School of Project Design, Tokyo, Japan; Department of Health Policy and Management, Nippon Medical School, Tokyo, Japan

**Keywords:** Self-reported sleep and energy, Reporting heterogeneity, Anchoring vignettes, World Health Survey, Japan

## Abstract

**Background:**

Anchoring vignettes are brief texts describing a hypothetical character who illustrates a certain fixed level of a trait under evaluation. This research uses vignettes to elucidate factors associated with sleep disorders in adult Japanese before and after adjustment for reporting heterogeneity in self-reports. This study also evaluates the need for adjusting for reporting heterogeneity in the management of sleep and energy related problems in Japan.

**Methods:**

We investigated a dataset of 1002 respondents aged 18 years and over from the Japanese World Health Survey, which collected information through face-to-face interview from 2002 to 2003. The ordered probit model and the Compound Hierarchical Ordered Probit (CHOPIT) model, which incorporated anchoring vignettes, were employed to estimate and compare associations of sleep and energy with socio-demographic and life-style factors before and after adjustment for differences in response category cut-points for each individual.

**Results:**

The prevalence of self-reported problems with sleep and energy was 53 %. Without correction of cut-point shifts, age, sex, and the number of comorbidities were significantly associated with a greater severity of sleep-related problems. After correction, age, the number of comorbidities, and regular exercise were significantly associated with a greater severity of sleep-related problems; sex was no longer a significant factor. Compared to the ordered probit model, the CHOPIT model provided two changes with a subtle difference in the magnitude of regression coefficients after correction for reporting heterogeneity.

**Conclusion:**

Sleep disorders are common in the general adult population of Japan. Correction for reporting heterogeneity using anchoring vignettes is not a necessary tool for proper management of sleep and energy related problems among Japanese adults. Older age, gender differences in communicating sleep-related problems, the presence of multiple morbidities, and regular exercise should be the focus of policies and clinical practice to improve sleep and energy management in Japan.

## Background

Sleep loss affects the cardiovascular, endocrine, immune, and nervous systems and leads to health problems such as obesity, diabetes, hypertension, anxiety, and depression [1]. Various dimensions of sleep disorders are associated with poor health outcomes. For example, poor self-reported sleep quality is associated with an increased risk of all-cause mortality, independent of sleep duration [2]. Self-reported short and/or long sleep duration and sleep disorders are associated with adverse health outcomes including hypercholesterolaemia, cardiovascular disease, obesity, diabetes, hypertension, depression and mortality [3–6]. Cumulative sleep deprivation may increase the risk of psychiatric disorders and accidents, while one night of insufficient sleep demonstrated decreased daytime cognitive function and driving performance [7]. In addition, difficulties initiating and maintaining sleep as well as early morning awakening are reported to have a close relationship with health dissatisfaction in adult populations from Japan, South Korea, and Taiwan [8].

Self-reported sleep-related problems have been associated with demographic, psychological, and factors related to health behavior in different settings. In the United States, sleep problems are more common in women and the elderly [9], and lower income and educational attainment are associated with more sleep disorders [10]. In Sweden, sleep-related problems are associated with being overweight (BMI > 27 kg/m^2^), physical inactivity, alcohol dependence, psychiatric disorders, and joint/low back disorders [11]. In addition, heavy binge drinking, smoking, unhealthy food habits, and physical inactivity were reported to be associated with trouble falling and staying asleep in Finland [12]. In Japan, sleep disorders are more prevalent in elderly populations [13]. Socio-economic status, measured according to employment type, is associated with sleep-related problems; male employees with more skilled occupations reported better sleep and health than those with less skilled occupations [14]. A study on Japanese adolescents revealed that sleep disorders are more prevalent among male junior high school students with poor mental health and who skip breakfast, drink alcohol, smoke, do not participate in extracurricular activities, and have late bedtimes [15]. However, the effects of important confounders such as morbidity due to angina, chest pain or discomfort when walking uphill or briskly, asthma, wheezing, or whistling breathing, feeling sad or empty, or depressed, or a loss of interest in most enjoyable things were never controlled for in studies on Japanese populations that examined factors associated with self-reported problems related to sleep quality. Therefore, elucidating factors associated with self-reported sleep quality while controlling for the aforementioned factors is needed.

**Fig. 1:**
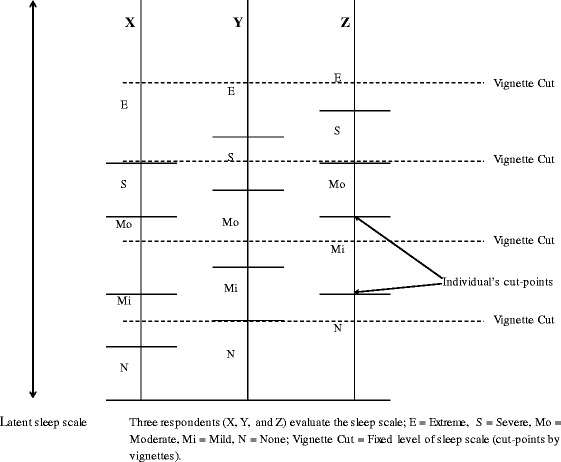
Hypothetical shifts in response category cut-points

Data obtained from self-reported health status is reported to be biased because people have different standards when assessing their health [16–18], i.e., people perceive their level of health differently. This perception is based on a latent, continuous scale of their health status and is translated into categorical responses; cut-points are determined based on personal backgrounds and experience (see Fig. 1). Cut-points define thresholds on a latent health scale, and individuals move from one response category to another. Failure to account for such reporting heterogeneity in self-assessment can potentially introduce biased estimates of a population’s health status. To overcome this problem, the approach of anchoring vignettes has been developed [19, 20]. Vignettes are hypothetical descriptions of various levels of health. They are brief, simple, and written in a culturally sensitive way to illustrate various levels of health using fictitious individuals. The anchoring vignettes approach is a promising tool and was suggested to address cut-point shifts in individual responses and improve intergroup comparability of self-reported measures [17, 18]. Although a number of studies have examined factors associated with self-reported problems related to sleep and energy in Japan [13–15], no study has attempted to test and adjust for reporting heterogeneity. Thus, the factors associated with self-reported sleep and energy in the general Japanese adult population were investigated while adjusting for aforementioned factors and correcting for reporting heterogeneity using anchoring vignettes. Through this process, we examined the potentiality of anchoring vignettes i.e., whether we need to correct for reporting heterogeneity in self-reported sleep and energy in Japan.

## Methods

### Data

Data were obtained from a pilot study of the World Health Survey, which was implemented by the World Health Organization from 2002 to 2004 in 70 countries to study adult health and health systems worldwide [21]. In Japan, the pilot study of the World Health Survey was conducted from 2002 to 2003 as part of a research project funded by the Ministry of Health, Labour and Welfare (principal investigator: Dr Toshihiko Hasegawa). The dataset is not publicly available and requires permission for use from the principal investigator as in the case of the current study. The objectives and methods of the pilot study are described elsewhere [22]. Briefly, the pilot study followed the methodologies of the World Health Survey, and the original questionnaires and instruments in English can be downloaded from the World Health Organization’s website [21]. These documents were translated into Japanese and then back-translated into English using the standard protocol of the World Health Organization. Five of 47 prefectures in Japan were chosen as study sites. These prefectures (Aomori, Tochigi, Shizuoka, Okayama, and Okinawa), stretching from the north to the south of Japan, were considered to geographically represent the whole country. Twenty-one municipalities that expressed willingness to participate in the survey were selected from both urban and rural areas of each prefecture. Households were randomly sampled from each municipality, all members aged ≥18 years of the sampled households were eligible for an interview, and 5016 respondents were surveyed. Trained interviewers visited households and surveyed respondents face to face. Among the 5016 person study sample, 1123 respondents were allocated to the vignettes survey. After excluding those with missing data for any variables used in the analysis, the final study sample size became 1002.

#### Measurements

##### Outcome variable

The outcome variable was generated from self-evaluations of the respondents’ sleep and energy related problems. The respondents were asked “On the whole, did you have any sleep-related problems such as difficulty falling asleep, waking up during the night or waking up too early and not being able to go back to sleep in the last 30 days?” The responses were based on a 5-point ordered categorical scale, ranging from 'none”, “mild”, “moderate”, “severe”, to “extreme”. The response categories were recoded as 1, 2, 3, 4, or 5, to represent the responses none, mild, moderate, severe, and extreme, respectively.

##### Sleep and energy related vignettes

There were five anchoring vignettes for sleep and energy, each representing a different severity level of sleep related problems (none/mild/moderate/severe/extreme) for hypothetical individuals. Table 1 shows the set of anchoring vignettes on sleep and energy related problems. To ensure response consistency, respondents were asked to rate the level of vignettes in the same way as they rated their own sleep and energy related problems. They were asked to assume that the age and socio-economic position of the hypothetical individuals described in the vignettes were similar to their own. The responses categories were based on a 5-point ordered categorical scale, ranging from none with a value of ‘1’, mild with a value of ‘2’, moderate with a value of ‘3’, severe with a value of ‘4’, and extreme with a value of ‘5’.

**Table 1:** Anchoring vignettes for sleep and energy in the Japanese World Health Survey

For each of the following situations, to what degree do you think that each person experienced sleep-related problems such as trouble falling asleep, waking up several times during the night or waking up too early in the morning during the last 30 days? Please mark as 1, None; 2, Mild; 3, Moderate; 4, Severe; 5, Extreme/cannot.
Vignette 1: It takes Mr./Ms. Aikawa less than five minutes from the time he/she gets into his/her bed until he/she falls asleep. He/she sleeps soundly and when he/she wakes up in the morning he/she feels that he/she has had a good night's sleep and is full of energy throughout the day.
Vignette 2: Mr./Ms. Ohyama has no difficulty falling asleep and does not wake up during the night but has a hard time getting up every morning. He/she uses an alarm clock but if he/she turns it off, he/she will fall back asleep. He/she is late four out of five days [a week] for work and feels drowsy all morning.
Vignette 3: Mr./Ms. Shindoh finds it easy to fall asleep, but twice a week he/she wakes up during the night and cannot sleep until morning. Recently, he/she is always exhausted at work and has difficulties concentrating on his/her job.
Vignette 4: Mr./Ms. Ohkawa generally wakes up every hour during the night. When he/she wakes up during the night, it usually takes him/her about 15 min to fall back to sleep. He/she does not feel as though he has had a good night’s sleep and feels listless and tired the whole day.
Vignette 5: It takes Mr./Ms. Ichise about two hours [starting] from the moment he/she gets into his/her bed to fall asleep. When he/she wakes up during the night he/she feels agitated and its takes him/her more than one hour to go back to sleep. Three or four times a week, he/she wakes up during the night and cannot sleep again until the morning. He/she is tired every day and all day long and misses work several times a week. He/she cannot play sports or participate in social activities.

Note: Names are included as examples only. Interviewers presented the set of names that matched the respondent’s gender; Vignette 1, 2, 3, 4, and 5 are none, mild, moderate, severe, extreme vignette, respectively

##### Explanatory variables

Independent variables included in the model were selected on the basis of existing literature [17, 18, 20, 22, 23]. These included single-year age, women (vs. men), years of schooling, number of morbidities, regular exercise, smoking, and alcohol use. The number of morbidities was generated from data on the diagnosis of seven available diseases and health problems, which included i) angina, ii) chest pain or discomfort when walking uphill or quickly, iii) asthma, iv) wheezing or whistling breathing, v) depression, vi) feeling sad, empty or depressed, and vii) a reported loss of interest in most enjoyable things. Respondents were grouped into categories representing having none, one, two, three, or more of these health conditions. A study based on data from individuals aged 18+ years from 60 countries in all regions of the world reported that depression comorbid with angina, asthma, arthritis and diabetes worsens overall health status [23]. Another study found associations between sleep problems and nine chronic conditions (angina, arthritis, asthma, chronic lung disease, depression, diabetes, hypertension, obesity, and stroke) for individuals aged 50+ years from China, Finland, Ghana, India, Mexico, Poland, Russia, South Africa and Spain [24]. An individual with any of the above seven diseases and health problems is thus assumed to have lower quality of health, and consequently the individual may have sleep-related problems. Regular exercise was recorded as “yes” or “no”, and current smokers were users of any tobacco product such as cigarettes, cigars, or pipe tobacco who responded “every day” or “yes, but not every day” to question on tobacco use. Those who responded, “I do not smoke” were considered non-smokers. Alcohol consumption was recorded as “yes” or “never” to the question, “Have you consumed any alcohol such as beer or wine?”

### Statistical analysis

In order to examine factors associated with sleep quality as well as correct for cut-point shifts in self-reported data, we conducted regression analyses to compare self-reported sleep quality with socio-demographic and lifestyle-related variables using the ordered probit model and the Compound Hierarchical Ordered Probit (CHOPIT) model. The CHOPIT model is an extension of the ordered probit model, which allows thresholds among response categories to vary as a function of individual characteristics. The CHOPIT model comprises two parts of the likelihood function: vignettes and self-assessments. The vignette component in the CHOPIT model uses ratings of the vignettes to estimate thresholds as a function of individual characteristics. The model has two assumptions about vignettes. First, irrespective of any characteristic, all respondents perceive the level of health represented in each vignette the same way, only assuming random measurement error (vignette equivalence). Second, respondents use the same response scale and set of cut-points to evaluate all vignettes and a self-assessment question for each domain (response consistency). The self-assessment component in the likelihood function only uses information on systematic reporting bias obtained from the vignette component. The self-reported ordered categorical data are mapped onto a latent continuous scale through the observation mechanism that is defined with the reporting thresholds estimated in the vignette component. This adapts respondents’ perceived level of health into an observed categorical response through the cut-points estimated from the vignette component. To apply the CHOPIT model, we adopted the generalized liner latent and mixed models (gllamm) program developed by Rabe-Hesketh and Skrondal [25]. All analyses were performed with STATA/SE 12.1 (StataCorp LP, College Station, TX, USA).

### Ethical considerations

This study was approved by the ethics review committee of the National Institute of Public Health in Japan. Informed consent was obtained from all respondents before each interview.

## Results

Table 2 shows the summary statistics of socio-demographic and lifestyle-related characteristics of the sample used in this analysis. The median age was 53 years, and more than half of the respondents (57 %) were female. Mean years of schooling was 12 years. About 69 % respondents did not have angina, chest pain or discomfort when walking uphill or quickly, asthma, wheezing or whistling breathing, depression, feeling sad, empty or depressed, and a reported loss of interest in most enjoyable things, while 5 % respondents had three or more of these diseases/health problems. About 42 % of respondents engaged in regular exercise, 23 % reported having a smoking habit, and 86 % reported regular use of alcohol. There were 19 (1.9 %), 96 (9.6 %), 158 (15.8 %), 260 (25.9 %), and 469 (46.8 %) participants reporting responses of extreme, severe, moderate, mild, and none, respectively, when questioned about their own sleep-related problems (results not shown).

**Table 2:** Socio-demographic and life-style characteristics of the study sample (*N* = 1002)

Characteristics	Value
Median age, years (range)	53 (18–91)
Female, no. (%)	576 (57.49)
Mean years of schooling, years (SD)	11.95 (2.66)
Number of morbidities, no. (%)	
None	689 (68.76)
One	178 (17.76)
Two	89 (8.88)
Three or more	46 (4.59)
Having no regular exercise, no. (%)	582 (58.08)
Smoker, no. (%)	230 (22.95)
Alcohol consumer, no. (%)	865 (86.33)

Note: *SD* standard deviation

Table 3 compares the results of regression analyses of self-reported sleep and energy with socio-demographic and life-style variables before and after correction for cut-point shifts. Older age and the presence of multiple morbidities were significantly associated with a greater severity of sleep-related problems in the ordered probit model. These associations remained statistically significant after correction for cut-point shifts in the CHOPIT model. Female gender was significantly associated with an increased severity of sleep-related problems in the ordered probit model, but this association was no longer significant in the CHOPIT model. There was no statistically significant association between regular exercise and sleep-related problems in the ordered probit model, while a lack of regular exercise was significantly associated with an increased severity of sleep-related problems in the CHOPIT model.

**Table 3:** Estimated regression coefficients of socio-demographic variables on self-reported sleep and energy before and after correcting for heterogeneity (*N* = 1002)

	Before correction		After correction	
	(Ordered probit model)		(CHOPIT model)	
	β	*p* value	β	*p* value
Age (years)	0.010	0.00	0.008	0.01
Women (vs. men)	0.161	0.05	0.107	0.27
Years of schooling	0.001	0.97	−0.026	0.16
Number of morbidities (reference: none)				
One	0.137	0.14	0.127	0.20
Two	0.646	0.00	0.650	0.00
Three or more	0.808	0.00	0.847	0.00
No regular exercise (vs. regular exercise)	0.104	0.16	0.194	0.03
Smoker (vs. non-smoker)	0.011	0.91	0.042	0.70
Alcohol consumer (vs. non-alcoholic)	0.108	0.32	0.082	0.53

Note: β regression coefficient; *CHOPIT* Compound Hierarchical Ordered Probit

No statistically significant associations between years of schooling, smoking, alcohol consumption and sleep-related problems were obtained in both ordered probit and CHOPIT models.

## Discussion

To the best of our knowledge, this is the first study in Japan to test and correct for reporting heterogeneity in an analysis of self-reported sleep and energy using anchoring vignettes. More than half of our study population reported having a mild or severe problem with sleep and energy. Before correcting for cut-point shifts using vignettes, older age, women, and the presence of comorbidities were significantly associated with a greater severity of sleep-related problems. After correction, the associations with age and the presence of comorbidities remained significant; however, the association with gender was no longer significant. Moreover, a lack of regular exercise was significantly associated with sleep-related problems. Therefore, age, comorbid health conditions, and exercise became significant predictors affecting self-reported sleep and energy after correction for reporting heterogeneity in the CHOPIT model. From the data used in the study, in the case of extreme vignettes for three dimensions of health (e.g. emotions, pain, sleep and energy), women were found to report severe health issues as being more problematic than do men (results not shown). This suggests that, for these dimensions, among Japanese women, health problems may be less severe than reported. This could be a potential reason behind the disappearance of the statistical significance of the association between gender and sleep-related problems when we correct for reporting heterogeneity in the CHOPIT model.

The vignette-based correction (CHOPIT model) provided two changes for women and regular exercise in the level of significance for self-evaluation of sleep and energy, but the direction of regression coefficients remained the same for both women and regular exercise. The differences in the magnitude of regression coefficients before and after correction for reporting heterogeneity were also subtle. Although anchoring vignettes are promising tools for the assessment of well-being and management of self-evaluation of health in different settings, our findings suggested that the ordered probit and the CHOPIT models produced similar results. Therefore, correction for reporting heterogeneity using anchoring vignettes for proper management of sleep and energy related problems is not suggested as a necessary tool for Japanese adults.

The prevalence of any self-reported sleep-related problems (extreme, severe, moderate, or mild) over the last 30 days was 53.2 %, while the prevalence of severe sleep-related problems (extreme or severe) was 11.5 %. Using pooled data from the 2002–2004 World Health Surveys of 57 countries (20 higher-income and 37 lower-income countries), which consisted of participants from all national income groups, a study showed that the prevalence of severe sleep-related problems (extreme or severe) for all countries combined was 7.6 %. For higher-income countries and lower-income countries, the prevalence was 7.8 % and 7.5 %, respectively [26]. Nakata and colleagues reported that 26.0 % of healthy Japanese male white-collar workers from an electric equipment manufacturing company had sleep-related problems [27]. In addition, Kaneita and others showed that 23.5 % of Japanese junior and senior high schools students have sleep-related problems [15]. Moreover, a study on a representative sample of the Japanese population reported that the prevalence of sleep problems experienced during the month preceding the survey was 21.4 % [28]. Although the aforementioned studies have examined the prevalence of sleep-related problems in Japan, their findings are not directly comparable to those of our study because they analyzed specific sub-groups over a different time frame, i.e., sleep-related problems were measured over one month.

Our results suggest that aging is associated with an increased severity of problems related to sleep and energy in Japan, and correction of reporting heterogeneity does not change this finding. Sleep disturbance is a common and complex clinical problem in older adults. An increase in the severity of sleep-related problems with age may be partly explained by an increase in the prevalence of diseases also associated with sleep and aging. A previous study found that complaints about sleep among older adults were associated with an increased number of respiratory and depressive symptoms, physical disabilities, use of nonprescription medications, and poor self-perceived health [29]. Thus, sleep-related problems in older adults reflect the presence of comorbid diseases and health conditions. These problems should be addressed in policy development and clinical practice to improve sleep quality in Japanese adults.

Women seem to have better sleep quality than men, with longer sleep time, shorter sleep-onset latency, and higher sleep efficiency. Despite this, women have more sleep-related complaints than men [30]. Gender-related differences in sleep disorders include differences in prevalence, pathophysiology, clinical presentation, and response to therapy [30]. Our results indicate that before correction for reporting heterogeneity, female gender was significantly associated with an increased severity of sleep-related problems; however, this association was no longer significant after correction. Based on the above discussion, rather than correcting for reporting heterogeneity using anchoring vignettes for sleep disorders, we recommend that doctors and other health care workers exercise caution when assessing the severity of sleep-related problems among Japanese women; sleep and energy problems may be less severe than reported among Japanese women.

Our study shows that the presence of comorbidities is associated with an increased severity of sleep-related problems, and after correction for reporting cut-point shifts our findings do not change. Common medical problems are often associated with sleep abnormalities. Patients who have chronic medical disorders tend to sleep fewer hours and experience less restorative sleep than those who do not, and insufficient sleep may worsen subjective symptoms of these disorders [31]. Therefore, treatment of comorbidities should be prioritized to properly manage sleep-related problems in Japan.

Our study suggests that a lack of regular exercise is associated with an increased severity of sleep-related problems after correction for reporting cut-point shifts. This finding is in agreement with those of previous studies. A previous study in Japan showed that sleep loss was associated with unhealthy lifestyles including a lack of exercise [32]. An epidemiological study also showed that regular exercise improves sleep quality [28]. Therefore, regular exercise should be promoted to enhance sleep and energy among Japanese adults.

### Limitations

This study has a few limitations. The dimensions of sleep-related problems (i.e., timing (intermittent/continuous) and duration) as well as details of lifestyle-related factors (i.e., exercise duration, the number of cigarettes smoked, and the amount of alcohol intake) could not be explored in this study because data on these factors were not available.

## Conclusions

Sleep-related problems are common among Japanese adults. Correction for reporting heterogeneity using anchoring vignettes is not a necessary tool for proper management of sleep and energy related problems among Japanese adults. When assessing sleep-related problems, healthcare providers and policymakers should carefully treat older adults, women, people with comorbidities, and those who do not exercise regularly in the development of health policies that affect clinical practice to improve sleep and energy management among Japanese adults.
